# Small ruminant lentiviruses in dairy goats under tropical conditions: evidence of recombinant genotypes

**DOI:** 10.1007/s11250-026-05069-8

**Published:** 2026-05-09

**Authors:** Nathalie Costa da Cunha, Alana de Oliveira Campello, Leandro dos Santos Machado, Thomas Salles Dias, Thalyta Rodrigues Silva, Arthur de Almeida Figueira, Beatriz Pinheiro Melo da Silva, Lilian Gregory, Barbara Colitti, Dayse Lima da Costa Abreu, Isadora de Fátima Braga Magalhães, Elmiro Rosendo do Nascimento, Mário Felipe Alvarez Balaro

**Affiliations:** 1 Department of Veterinary Collective Health and Public Health, Fluminense Federal University (UFF), Niterói, Rio de Janeiro, Brazil; 2 Department of Epidemiology and Public Health, Federal Rural University of Rio de Janeiro (UFRRJ), Seropédica, Rio de Janeiro, Brazil; 3 Department of Internal Medicine, School of Veterinary Medicine and Animal Science, University of São Paulo (USP), São Paulo, Brazil; 4 Department of Veterinary Sciences, University of Turin, Largo Paolo Braccini 2, 10095 Grugliasco, Italy; 5 Department of Veterinary Pathology and Clinical Veterinary Medicine, Fluminense Federal University (UFF), Niterói, Rio de Janeiro, Brazil

**Keywords:** Lentivirus, Goats, SRLV, Recombination, Phylogenetics, Prevalence, Brazil

## Abstract

Small Ruminant Lentiviruses (SRLVs) are widespread retroviruses of goats and sheep with significant economic and health impacts on small ruminant production systems. This study aimed to investigate the prevalence and genotype distribution of SRLVs in dairy goat flocks from Brazil. A total of 433 blood samples from eight dairy goat farms were analyzed via PCR targeting the *gag* gene. The overall SRLV prevalence was 32.6%, with marked variation between farms (7.1% to 90.0%). Phylogenetic analysis of 37 selected samples revealed that most sequences clustered within the B1 subtype. Notably, two sequences from farms C and G grouped with subtype A1 and showed evidence of recombination between B1 and A1 strains. These findings provide evidence of recombinant SRLV strains circulating in Brazilian goats. The findings highlight the circulation of distinct and recombinant SRLV strains in the region, reinforcing the importance of continuous molecular surveillance, improved biosecurity practices, and the development of effective control strategies to reduce viral dissemination and mitigate long-term impacts on small ruminant farming.

**Supplementary Information:**

The online version contains supplementary material available at 10.1007/s11250-026-05069-8.

## Introduction

Caprine and ovine farming is an economic activity exploited on all continents, even in ecosystems that are highly distinct in terms of climate, soil, topography, and vegetation (Silva et al. 2022). Initially in Brazil, this production was a subsistence activity to complement cattle raising. Goats and sheep were raised to feed cattle breeders, as their value was much lower than that of other livestock. It was not seen as a dynamic and modern sector, so the animals were raised extensively, with low technology, and their results were minimal (Callado et al. 2001; Gonçalves et al. 2008). The predominant focus of goat farming varies by country (Miller and Lu 2019; Silva et al. 2022; Nguyen et al. 2023), with an emphasis on milk production in many regions, although some breeds are also valued for meat. For instance, Brazil stands out in Latin America and Caribbean as the leading producer of goat milk (Villarreal-Ornelas et al. 2022).

Proper sanitary management is essential for flock maintenance. The activity requires technical assistance, yet there are still many breeders who neglect simple and fundamental practices (Rodrigues et al. 2017). Diseases caused by Small Ruminant Lentiviruses (SRLV) can affect animals of all ages, regardless of gender and breed, and are usually referred to as Caprine Arthritis-Encephalitis (CAE) and Maedi-Visna (MVV) (Olech 2023). These viruses have a global distribution and are mandatorily notifiable to the World Organisation for Animal Health due to their significant capacity for dispersion, economic and/or social consequences, serious health impacts, and national and international repercussions (WOAH 2023).

SRLV belongs to four distinct phylogenetic groups and possesses numerous subtypes, which exhibit genetic similarity, molecular replication, morphology, and host interaction. This classification is achieved through molecular analysis of proviral DNA determined by targeting the different viral genes, such as *gag*–*pol* regions (Shah et al. 2004; Bouzalas et al. 2024). While SRLV agents were traditionally regarded as species-specific, different studies suggest natural cross-species transmission (Arcangeli et al. 2022).

In Brazil, few studies have investigated the prevalence of Small Ruminant Lentiviruses using molecular methods and even fewer phylogenetic analyses. So, the present study aimed to investigate the prevalence and the circulating genotypes of SRLV from flocks **i**n the state of Rio de Janeiro using samples received by the Laboratory of Molecular Epidemiology-UFF between 2016 and 2024.

## Materials and methods

### Sampling

A convenience sampling approach was used, based on blood samples submitted for diagnostic purposes to the Veterinary Diagnostic and Epidemiology Service of the Fluminense Federal University (UFF) between 2016 and 2024. The years 2020 and 2021 were excluded from this report due to the COVID-19 pandemic.

Samples were obtained from a subset of the herd, including both asymptomatic goats and symptomatic animals suspected of SRLV infection, presenting clinical signs such as weight loss, arthritis, and/or mastitis.

### DNA Extraction and SRLV proviral amplification

The samples were subjected to DNA extraction from buffy coat using the Wizard^®^ Genomic DNA Extraction Kit (Promega™, Brazil), following the manufacturer’s recommendations. The obtained DNA was quantified using the Nanodrop spectrophotometer (Biodrop™) and stored at -20 °C until molecular analysis.

The diagnosis of SRLV infection was made through polymerase chain reaction (PCR) with specific primers for the *gag* gene according to Barlough et al. (1994). Amplification products were confirmed by electrophoresis on 1.5% agarose gel stained with ethidium bromide by detection of a 187-bp fragment.

### SRLV genotyping

All PCR-positive samples were submitted to sequencing. Of these, 37 samples with high-quality sequences were selected for further phylogenetic analysis. An 800 bp *gag* gene fragment was amplified and sequenced according to Grego et al. (2007). Briefly, the first round of PCR was conducted in a reaction volume of 50 µl, using 10 µl of DNA sample, 1X PCR buffer, 1.5 mM MgCl2, 0.3 µM of each primer, 0.2 µM of dNTP, and 1U of GoTaq^®^ Hot Start Taq Polymerase (Promega™, Brazil). The second round of PCR was performed under the same conditions as the first round, using 4 µl of the first PCR product as template.

All positive samples on the agarose gel that showed the band of the expected size have been excised and were purified using the commercial Wizard^®^ Genomic DNA Purification Kit, following the manufacturer’s instructions. The purified products were then subjected to the Sanger sequencing method at the Fundação Oswaldo Cruz Sequencing Platform. The final sequences used for phylogenetic analysis varied in length, ranging from 120 to 781 bp, and were deposited in GenBank under accession numbers PX216601–PX216637.

### Data analysis

Confidence intervals (95% CI) for SRLV prevalence were calculated using the binomial approximation method (Wald method).The sequences were aligned using ClustalW (Thompson 1997) with 93 reference sequences available in GenBank. Phylogenetic trees were constructed using MrBayes ver. 3.1.1 (Ronquist and Huelsenbeck 2003), considering a molecular evolution model estimated by MODELTEST ver. 3.7 (Posada and Crandall 2001). Tree statistics and phylogenetic manipulations were conducted using the computer program PAUP* ver. 4.0b10 (Swofford and Sullivan 2009). Genetic diversity was expressed as nucleotide diversity (Nei 1987). Recombination analysis was performed using RDP4 software ver. 4.101, applying multiple detection methods (RDP, GENECONV, BootScan, MaxChi, Chimaera, SiScan, and 3Seq) with Bonferroni correction for multiple comparisons (*p* < 0.05). Breakpoints were inferred using default parameters, and recombination events were considered credible when supported by at least three independent methods.

## Results

**Fig. 1:**
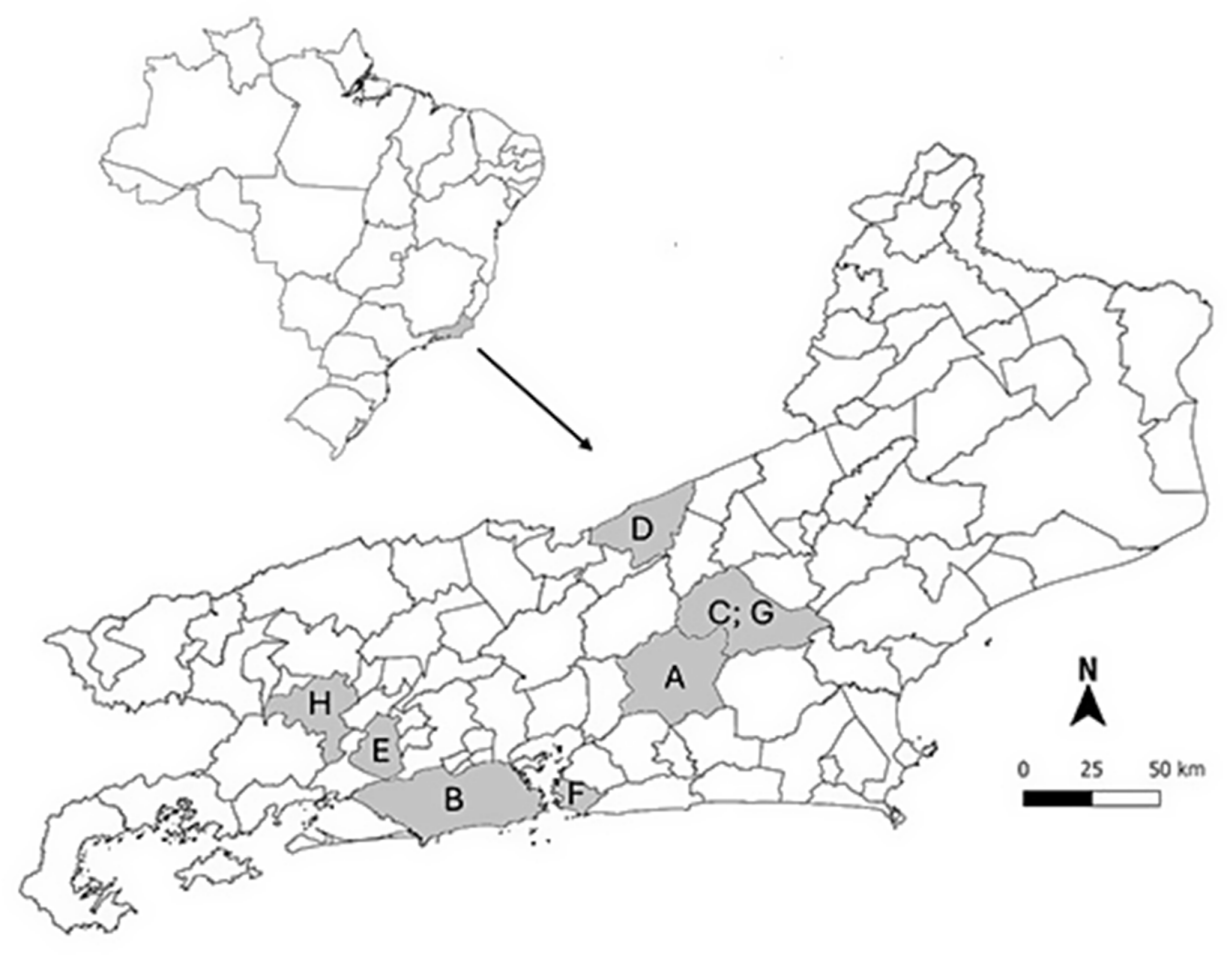
Map of the municipalities of Rio de Janeiro state, Brazil, highlighting the studied flocks, from A to H

This study investigated the prevalence of Small Ruminant Lentivirus (SRLV) infection in dairy goat flocks across Rio de Janeiro state. A total of 8 dairy goat flocks (labeled A–H) and 433 animals were sampled, and the number of samples, number of positive cases, and corresponding positivity percentages were analyzed. The overall SRLV prevalence was 32.6% (141/433; 95% CI: 28.2–37.0%), in the period from 2016 to 2024, excluding the years 2020 and 2021. All flocks were positive. The results are summarized in Table 1 and Supplementary Table S1. Figure 1 presents the municipalities of the flocks studied in the state of Rio de Janeiro.

**Table 1:** Prevalence of SRLV infection, number of samples sequenced, and genotypes identified in dairy goat herds from Rio de Janeiro State, Brazil

Flock	Number of positive samples/Number of tested samples	Prevalence % (95% CI)	Samples sequenced	SRLV genotype(s) identified
A	10/107	9.3 (3.8–14.9)	--	--
B	1/14	7.1 (0.0–20.6)	--	--
C	18/20	90.0 (76.8–100.0)	9	B1; B1/A1 recombinant
D	48/89	53.9 (43.6–64.3)	9	B1
E	8/51	15.7 (5.7–25.7)	5	B1
F	17/47	36.2 (22.4–49.9)	3	B1
G	34/59	57.6 (45.0–70.2)	11	B1; B1/A1 recombinant
H	5/46	10.9 (1.9–19.9)	--	--
**Total**	**141/433**	32.6 **(28.2–37.0)**	**-**	**-**

-- No sequences available

Among the flocks studied, Flock A, located in Rio de Janeiro (RJ), had the largest sample size (107 samples), with 10 positive cases, resulting in a 9.3% positivity rate. Flock C, which showed the highest SRLV prevalence, had 18 positives out of 20 samples, corresponding to a 90.0% positivity rate. Flock D also had a notable prevalence of 53.9%. Flocks A, B, and H showed relatively low prevalence, with positive rates of 9.3%, 7.1%, and 10.9%, respectively.

**Fig. 2:**
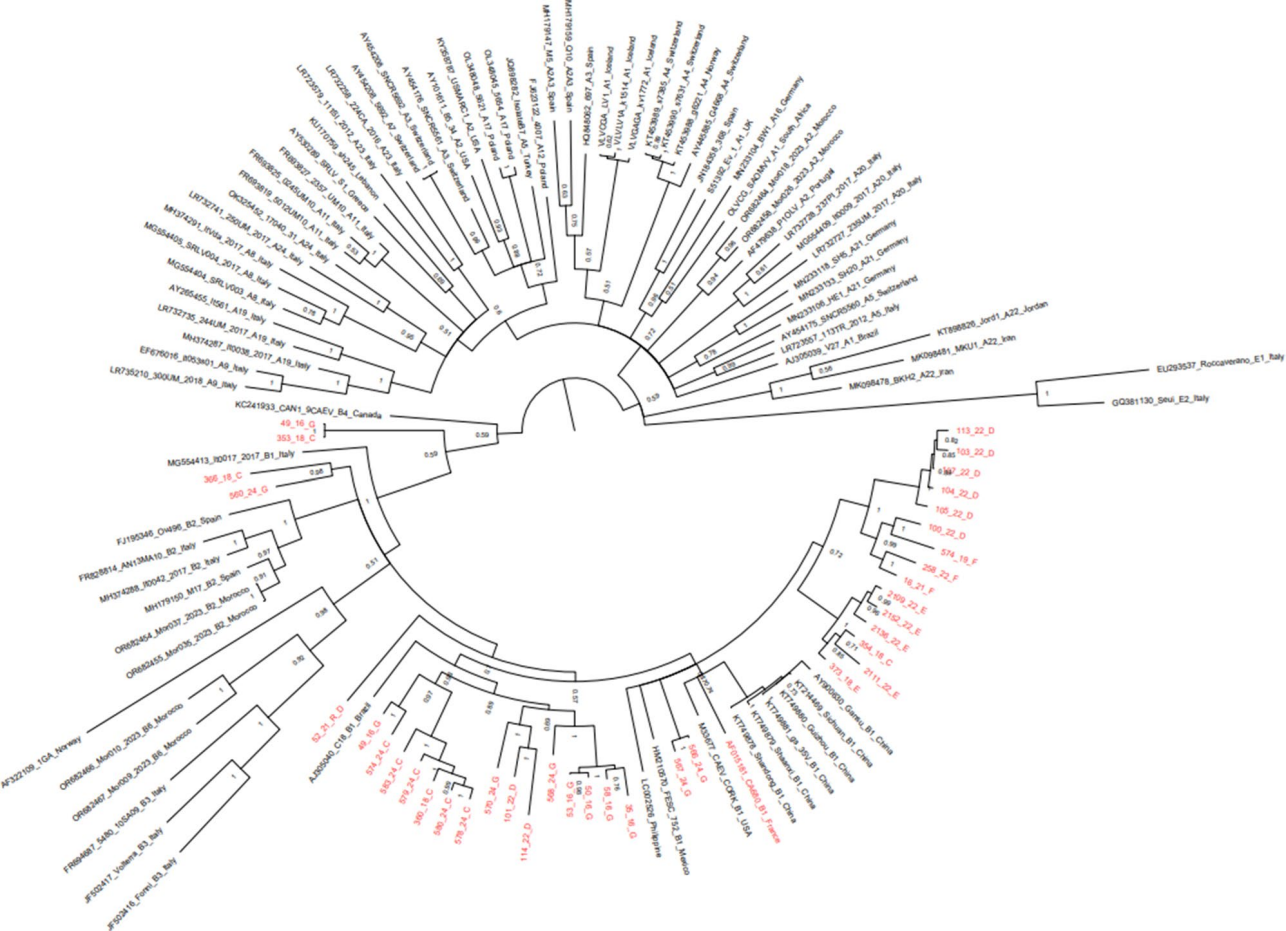
Phylogenetic tree obtained from the alignment of the 37 Brazilian sequences and 93 reference strains retrieved from the GenBank database. Reference sequences are reported with the country of isolation and subtype if available. Newly characterized sequences are reported in red. Posterior probability of each node is shown above the branches

Thirty-seven out of 141 positive samples were successfully sequenced by Sanger methodology and included in the subsequent phylogenetic analyses. Phylogenetic reconstruction showed that the majority of Brazilian sequences clustered within the B1 subtype (Fig. 2). Notably, two sequences from farms C and G (49_16, GenBank accession number PX216621; and 353_18, GenBank accession number PX216620) grouped within the A subtype clade, displaying mean nucleotide identities of 84.88% with a previously described Brazilian A1 strain and 83.18% with B1 subtype strains (Supplementary Table S2). Recombination analysis further demonstrated that these two sequences represent recombinant strains, characterized by a mosaic genomic structure resulting from recombination between A1 and B1 parental lineages.

**Fig. 3:**
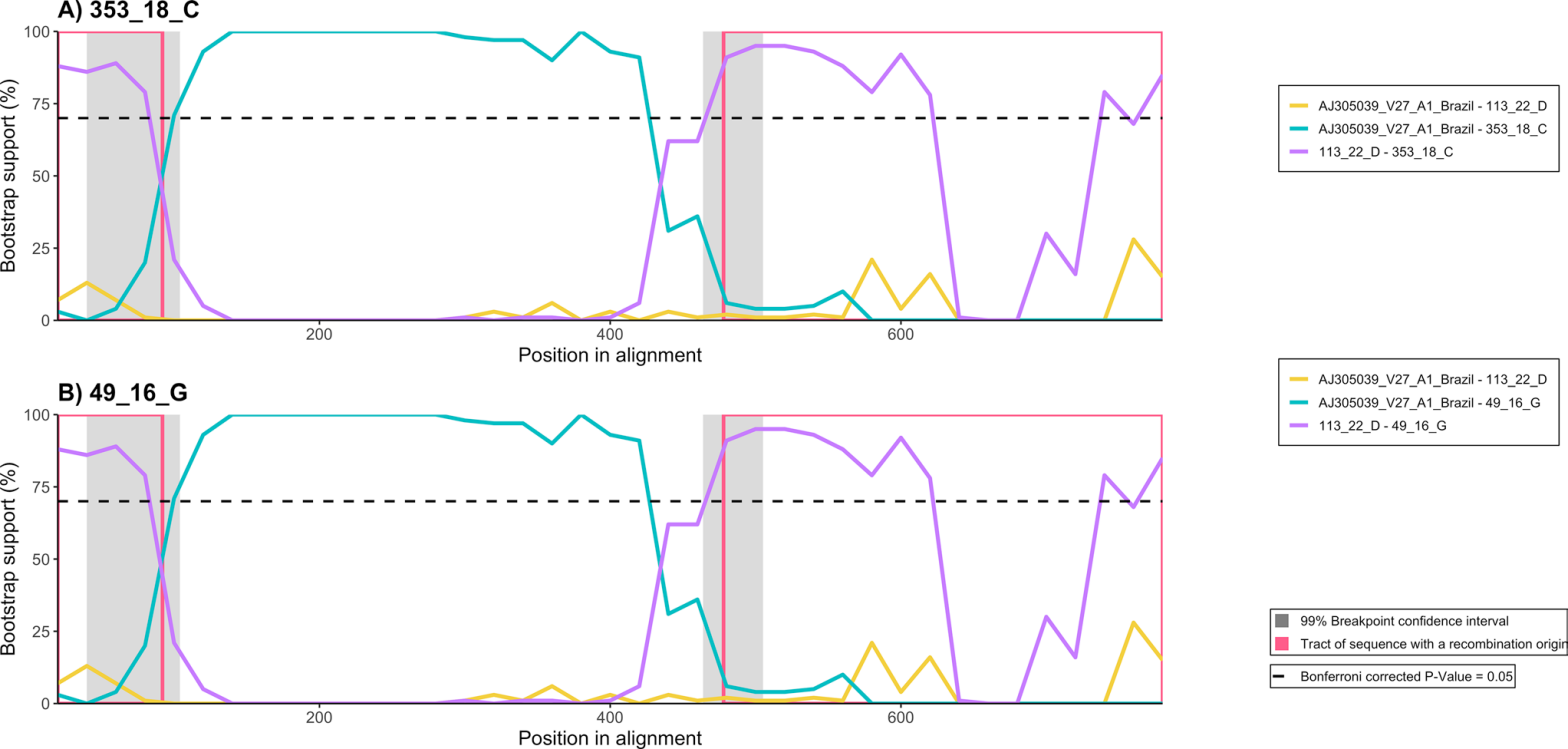
Recombination analysis performed using RDP4 v.4.101 software. Bootscanning plot of the *gag r*egion from sequences 49_16 (**A**) and 353_18 (**B**), amplified from animals belonging to farms G and C, respectively. The y-axis represents bootstrap support (%) for clustering with the inferred parental sequences along the alignment (x-axis). Regions of recombinant origin are indicated by pink brackets. The dashed horizontal line indicates the significance threshold (Bonferroni-corrected *p* = 0.05). Grey shaded areas represent the 99% confidence intervals for the inferred recombination breakpoints

Recombination analysis performed using RDP4 identified a significant recombination event involving strains 49_16 (Accession number PX216621) and 353_18 (Accession number PX216620). The two sequences showed a B1/A1 pattern, representing recombinant sequences between V27 A1 Brazilian strain (Accession number AJ305039) and the newly characterized 113_22 B1 strain (Accession number PX216612) from farm D, as shown in Fig. 3. The event was supported by five independent detection methods (RDP, BootScan, MaxChi, Chimaera, and 3Seq), with Bonferroni-corrected p-values < 0.01, indicating strong statistical support. The inferred recombinant fragment spans positions 93 to 478 of the alignment, with breakpoint confidence intervals of 40–104 and 464–505 (99% CI).

BootScan analysis revealed a clear phylogenetic shift across the breakpoint regions, consistent with a mosaic genome structure. The average bootstrap support for the recombinant signal was 77.5% for strain 49_18 and 68.3% for strain 353_18, suggesting stronger phylogenetic support. Overall, the concordant detection across multiple independent methods, together with statistically significant p-values and defined breakpoint intervals, supports the occurrence of a genuine recombination event in both strains.

**Fig. 4:**
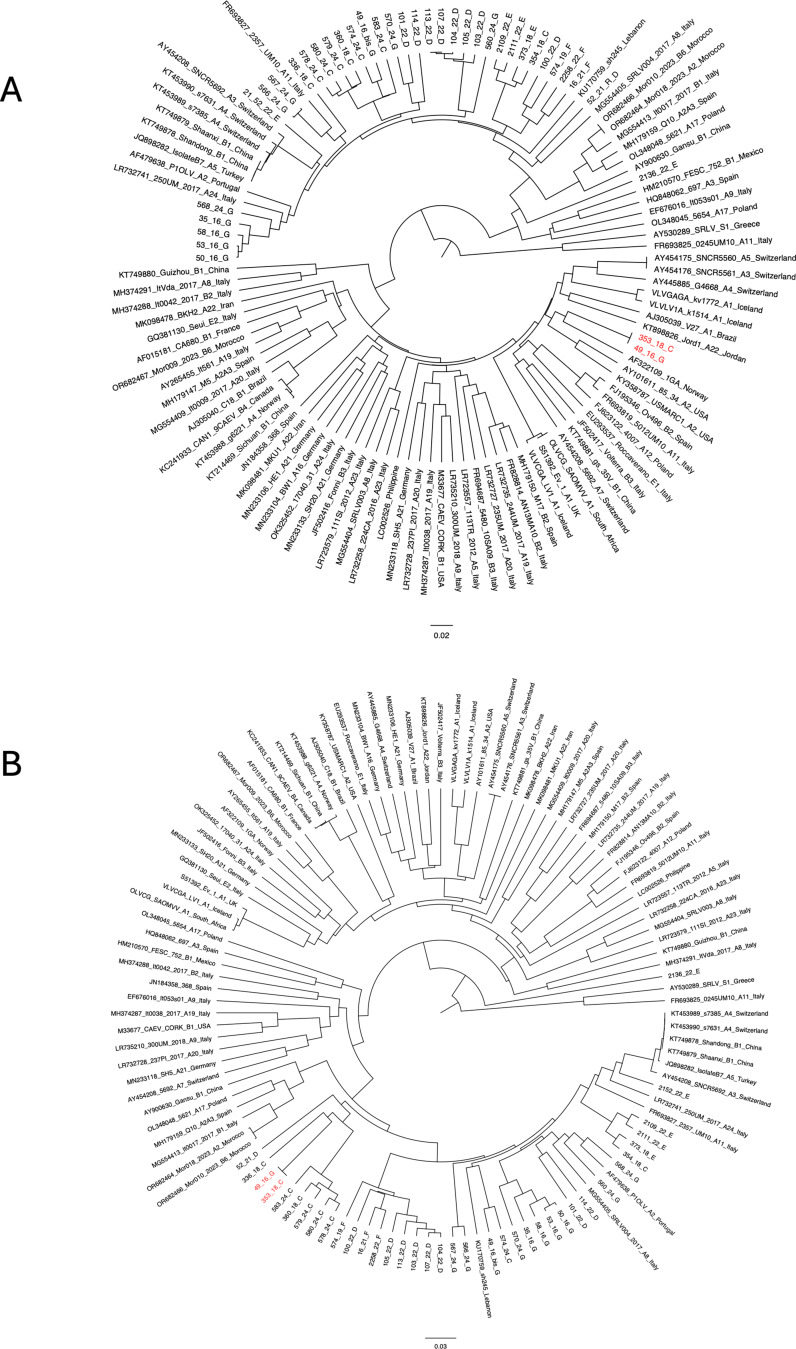
UPGMA trees showing phylogenetic relationships of the newly characterized recombinant strains 49_16 (GenBank accession number PX216621) and 353_18 (GenBank accession number PX216620) (highlighted in red) and reference strains from major parent (position 93 to 478) **(A**) and minor parent (position 1 to 92 and 479 to 784) (**B**). Bootstrap values were calculated from 1000 replicates

The recombination event detected by RDP4 was confirmed considering the UPGMA trees based on 1000 bootstrap replicates obtained by splitting the 784 bp alignment into multiple new alignments based on detected breakpoint positions, as shown in Fig. 4. The tree derived from the major parent in position 93–478 showed that the two sequences 49_16 (GenBank accession number PX216621) and 353_18 (GenBank accession number PX216620) clustered with the A1 strains (with 77% bootstrap support), while the tree derived from the minor parent in position 1–92 and 479–784 showed that the sequences clustered with the B1 strains (with 78% bootstrap support). This statistically supported phylogenetic incongruence across genomic regions provides independent confirmation of the mosaic genome structure.

## Discussion

In this study, the prevalence of Small Ruminant Lentivirus (SRLV) infection in dairy goat flocks has been investigated in 8 dairy goat flocks across Rio de Janeiro state using a molecular-based approach. The results showed that SRLV is widespread in the flocks sampled in Rio de Janeiro state, as previously reported in Brazil, even if a higher prevalence of positive animals was recorded when compared to other studies from Brazil (Guilherme et al. 2017; Rodrigues et al. 2017; Teles et al. 2023). However, the differences in recorded prevalences can be explained considering that most of these studies used serology in the screening of SRLV prevalence. The Agarose Gel Immunodiffusion technique is widely used for diagnosing infected animals due to its cost-effectiveness and straightforwardness. However, some animals may have low levels of antibodies, delayed immune responses, or inconsistent test results, which should be considered during routine diagnosis (Marinho et al. 2018). PCR is a widely documented technique used for detecting CAEV infection (Ravazzolo et al. 2001; Rodrigues et al. 2017; Furtado Araújo et al. 2020) by amplifying specific regions of the viral genome, PCR enables the sensitive and specific identification of CAEV nucleic acids within clinical samples, such as blood or tissue specimens. Its high sensitivity allows for the detection of even low viral loads, facilitating early diagnosis. Given the nature of retroviral infections and their complex interaction with the host immune system, combining serological and molecular techniques can enhance diagnostic accuracy and improve surveillance efforts (Olech 2023).

Based on these findings, the variation in SRLV prevalence among flocks in this study may be explained by differences in herd size, management practices, biosecurity measures, and animal movement. Higher-prevalence flocks, such as C and D, more frequently introduced new animals without quarantine or prior testing for SRLV, whereas lower-prevalence flocks, such as A, B, and H, showed lower animal circulation and stricter management practices. The herds analyzed in Rio de Janeiro are located in a tropical region (Neiva et al. 2017) and were characterized by intensive or semi-intensive dairy production systems, predominantly involving Saanen goats, where the introduction of animals from external sources and the absence of strict quarantine measures may facilitate viral dissemination within and between herds. Similar risk factors have been reported in other studies. In Portugal, larger and intensively managed goat flocks and those introducing replacement animals were more likely to be infected with SRLV (Jacob-Ferreira et al. 2023). Likewise, in Brazil, importing animals from other states, not isolating sick animals, and participation in fairs were associated with higher SRLV prevalence, highlighting the role of herd management and animal movement in SRLV transmission dynamics (Teles et al. 2023).

Phylogenetic analysis revealed that most Brazilian SRLV sequences clustered within the B1 subtype, consistent with previous reports of subtype B1 dominance in Latin America (Ramírez et al. 2011; Hasegawa et al. 2016; De la Luz-Armendáriz et al. 2021). Beyond subtype assignment, phylogenetic topology also provides insights into the regional and global circulation patterns of SRLV B1 strains. In this study, the Brazilian B1 clusters are interspersed with B1 strains from other Latin American countries, particularly Mexico, and with strains from Europe. This pattern is consistent with what has been described for subtype B1 globally (Ramírez et al. 2011; Hasegawa et al. 2016; De la Luz-Armendáriz et al. 2021; Arcangeli et al. 2022), which shows low phylogeographic structure and wide international mixing, largely attributed to historical and ongoing animal movement and trade. Therefore, the topology suggests regional circulation within Latin America, but not exclusive regional evolution. The proximity of Brazilian B1 sequences to Mexican and other Latin American strains supports the idea that B1 has been disseminated across the region through shared production systems, animal exchange, and importation of breeding stock, rather than evolving independently within Brazil. At the same time, the absence of a monophyletic Brazilian clade argues against long-term local diversification in isolation. Instead, the Brazilian strains appear to be part of a broader, globally circulating B1 pool, which aligns with previous reports describing subtype B1 as the most cosmopolitan SRLV subtype.

Two sequences (from farms C and G) clustered with the A subtype, showing a mean sequence identity of 84.88% to a previously reported Brazilian A1 strain and 83.18% to B1 subtype strains. Subtype A1 has been previously identified in small ruminants in Brazil, where both A1 and B1 subtypes co-circulate in goats and sheep, indicating natural interspecies transmission and high genetic diversity within the country’s SRLV population (Braz et al. 2022). The presence of A1 alongside B1 in the same host populations is consistent with global reports of SRLV genotype A variants infecting goats, where multiple subtypes such as A1 have been documented across different regions and host species (Olech and Kuźmak 2024). This divergence between the A1-like sequences and B1 strains reflects circulating subtype heterogeneity rather than simple sequencing error, supporting the notion that distinct SRLV subtypes are maintained in Brazilian goat populations. The co-occurrence of divergent subtypes may contribute to variable clinical outcomes and complex transmission dynamics, as observed in other SRLV molecular epidemiology studies that highlight the influence of subtype diversity on infection persistence, host tropism, and control challenges (Ramírez et al. 2013).

Recombination events were identified in two sequences through RDP4 analysis, highlighting the genetic plasticity of SRLV. These sequences exhibited a B1/A1 recombinant pattern, involving the V27 A1 strain (Accession: AJ305039) and a newly characterized B1 strain (113_22) from farm D. The recombinant origin was further confirmed through phylogenetic analyses of the *gag* gene, which showed clustering with A1 strains in the major parent region (positions 93–478) and with B1 strains in the minor parent regions (positions 1–92 and 479–784). These findings suggest that the circulation of Small Ruminant Lentiviruses in Brazil can involve more variable strains, highlighting the importance of monitoring their genetic heterogeneity through molecular and serological strategies. Recombination in small ruminant lentiviruses has been reported in different regions of the world, reflecting the high genetic plasticity of these viruses (L’Homme et al. 2015; Olech and Kuźmak 2021). In this context, our results provide evidence of recombinant SRLV strains circulating in dairy goat herds in Brazil, representing the first report of SRLV recombination in goats in the country. Consistent with reports from Canada (L’Homme et al. 2015), co-infection with A-group and B-group strains has led to recombination events, resulting in mosaic strains that are now circulating among goat populations in the country. The presence of recombinant strains is of particular significance as recombination is a known mechanism for viral evolution, potentially leading to altered pathogenicity, immune evasion, or host adaptability (Olech and Kuźmak 2021). The identification of recombination sites in SRLV genomes underscores the need for ongoing genomic surveillance to detect emerging variants with enhanced virulence or transmissibility. The detection of recombinant SRLV strains also has relevant implications for disease control. Recombination contributes to viral genetic diversity and may influence transmission dynamics and the performance of diagnostic tools. Therefore, the identification of recombinant viruses reinforces the importance of continuous molecular surveillance to better understand SRLV evolution and to support effective control strategies in goat populations (L’Homme et al. 2015; Olech and Kuźmak 2021).

One limitation of this study is that the samples originated from diagnostic submissions from herds with clinical signs compatible with SRLV infection rather than from random sampling. Therefore, the reported prevalence should be interpreted with caution, as it may overestimate the true prevalence in the regional population. Nevertheless, the findings confirm SRLV circulation and provide relevant information on circulating genotypes, including the detection of recombinant strains in Brazil.

The findings of this study provide important information for veterinarians and goat producers by highlighting the circulation of genetically diverse SRLV strains in dairy goat herds from Rio de Janeiro state. The detection of recombinant viruses reinforces the importance of molecular surveillance and the implementation of appropriate biosecurity measures, particularly regarding animal movement and herd replacement practices.

## Conclusion

This study highlights the prevalence and molecular characteristics of SRLV infections across dairy goat flocks in Rio de Janeiro state, Brazil. The findings reveal significant variation in SRLV prevalence among flocks, with rates ranging from 7.1% to 90.0%. Most sequences belonged to the B1 subtype, while two sequences from farms C and G exhibited recombination between the B1 and A1 subtypes. This recombination event highlights the genetic complexity and potential for viral evolution within the region. These findings emphasize the need for ongoing surveillance and molecular characterization of SRLV to better understand its epidemiology and genetic diversity. The high prevalence in some flocks, coupled with the detection of recombinant strains, underscores the importance of implementing control strategies and monitoring programs to mitigate the spread and impact of SRLV infections in Brazilian goat populations.

## Supplementary Information

Below is the link to the electronic supplementary material.

Supplementary Material 1

Supplementary Material 2

## Data Availability

The data that support the findings of this study are available from the corresponding author upon a reasonable request.
